# Draft Genome Sequences of Nitrogen-Fixing Bradyrhizobia Isolated from Root Nodules of Peanut, Arachis hypogaea, Cultivated in Southern Tunisia

**DOI:** 10.1128/MRA.00434-21

**Published:** 2021-07-22

**Authors:** Besma Bouznif, Benoit Alunni, Mohamed Mars, Jacqui A. Shykoff, Tatiana Timchenko, Ricardo C. Rodríguez de la Vega

**Affiliations:** a Université Paris-Saclay, CNRS, AgroParisTech, Ecologie Systématique et Evolution, Orsay, France; b Université Paris-Saclay, CEA, CNRS, Institute for Integrative Biology of the Cell (I2BC), Gif-sur-Yvette, France; c Laboratory of Biodiversity and Valorization of Arid Areas Bioresources (BVBAA), Faculty of Sciences, Gabès, Tunisia; University of Arizona

## Abstract

Here, we report the draft genome sequences of two nitrogen-fixing symbionts, *Bradyrhizobium* sp. strain sGM-13 and *Bradyrhizobium* sp. strain sBnM-33, isolated from root nodules of peanut grown on soil samples collected from two regions in South Tunisia. The draft genome sizes of these two strains are 8.31 × 10^6^ bp and 8.97 × 10^6^ bp, respectively.

## ANNOUNCEMENT

Nitrogen-fixing bacteria called rhizobia are known for their ability to form symbioses with a wide range of legumes and convert atmospheric nitrogen into a form that can be assimilated by plants (1). Peanut, Arachis hypogaea, is an important oilseed legume originally from South America. Peanut is now cultivated worldwide and forms effective nodules mainly with slow-growing bacteria belonging to the genus *Bradyrhizobium* (2–5). The study of the diversity of symbiotic bacteria nodulating peanut provides a basis for the development of rhizobial inoculants well adapted to the environmental conditions of the local areas of cultivation, which subsequently alleviate the use of synthetic fertilizers (4, 6, 7).

In this study, we report the draft genome sequences of two strains, *Bradyrhizobium* sp. strain sGM-13 and *Bradyrhizobium* sp. strain sBnM-33, symbionts of Arachis hypogaea. As a first step, we collected soil samples from two sites in southern Tunisia, one approximately 8 km south of Ben Gardane (33°05′N, 11°14′E) and one approximately 10 km south of Gabès (33°48′N, 10°08′E). Then, we trapped nodulating bacteria from pots containing these soils planted with local varieties of peanut. After cultivation for 7 weeks, plants were harvested and nodules were collected from the roots. Then, the nodules were surface sterilized, crushed, and streaked onto yeast mannitol agar (YMA) medium (8) to isolate the bacteria inside the nodules. The strains isolated were checked for their ability to renodulate their host plant, Arachis hypogaea. The isolated strains were identified as *Bradyrhizobium* based on partial sequencing of the 16S rRNA gene using the primers PA (AGAGTTTGATCCTGGCTCAG) and PH (AAGGAGGTGATCCAGCCGCA) (expected insert size, 1,500 bp) (9).

A single colony of each strain was grown in liquid yeast extract mannitol (YEM) medium for 4 days at 28°C. Then, the total genomic DNA was extracted from the two strains using the MasterPure complete DNA and RNA purification kit (Epicentre). Genomic DNA was fragmented by sonication, and fragments were polished, A-tailed, multiplexed, and ligated to Illumina adapters. Libraries were purified with an AMPure XP system and paired-end sequenced in a NovaSeq instrument (library preparation and sequencing subcontracted to Novogene, United Kingdom). Paired-end sequencing generated between 4.4 and 4.5 million reads of 2 × 150 bp. The reads were trimmed and filtered using Trimmomatic version 0.22 (10) with the options ILUMINACLIP:TrueSeq3-PE-2:3:30:10 HEADCROP:6 LEADING:30 TRAILING:30 SLIDINGWINDOW:4:30. Assemblies were then obtained with the SPAdes genome assembler version 3.11.1 (11) using the careful mode. Annotation of the *Bradyrhizobium* sp. sGM-13 and *Bradyrhizobium* sp. sBnM-33 genome sequences was performed using Prokka (12). Coverage was calculated with SAMtools mpileup (13) on Burrows-Wheeler Aligner MEM algorithm (BWA-MEM) version 2-generated (14) mapping of the paired-end reads. Information about the two draft genome sequences is listed in Table 1.

**TABLE 1:** Characterization of draft genome sequences of *Bradyrhizobium* sp. sGM-13 and *Bradyrhizobium* sp. sBnM-33

Characteristic	Data for:	
	*Bradyrhizobium* sp. sGM-13	*Bradyrhizobium* sp. sBnM-33
No. of reads	4,549,384	4,435,615
No. of contigs >200 bp	574	330
No. of genes	7,906	8,678
Draft genome length (bp)	8,309,700	8,966,052
*N*_50_ (bp)	247,668	121,392
G+C content (%)	62	62
Coverage (×)	129	117
BioSample accession no.	SAMN18828746	SAMN18828747
SRA accession no.	SRR14380225	SRR14380224
Assembly accession no.	JAGWDK000000000	JAGWDJ000000000

The strains sGM-13 and sBnM-33 had average nucleotide identities (ANI; calculated over all coding sequences [CDS] as in https://img.jgi.doe.gov/docs/ANI.pdf) of 90.99% and 88.60%, respectively, in relation to the closest species, Bradyrhizobium retamae Ro19 (GenBank accession no. NZ_LLYA00000000.1) and 89.53% with respect to each other. These values are well below the generally accepted species threshold of 95%, and therefore, they appear to belong to two distinct species (15, 16). These two strains found in the arid soils of southern Tunisia present promising raw materials for the development of bacterial inoculum to improve peanut cultivation in the southern Mediterranean region.

### Data availability.

The raw reads and draft contigs of *Bradyrhizobium* sp. sGM-13 and *Bradyrhizobium* sp. sBnM-33 were deposited at GenBank under the BioProject no. PRJNA723783. The BioSample numbers, raw reads, and assembly GenBank accession numbers are given in Table 1.

## References

[1] Mylona P, Pawlowski K, Bisseling T. 1995. Symbiotic nitrogen fixation. Plant Cell 7:869–885. doi:10.1105/tpc.7.7.869. 12242391 PMC160880

[2] Muñoz V, Ibañez F, Tonelli ML, Valetti L, Anzuay MS, Fabra A. 2011. Phenotypic and phylogenetic characterization of native peanut *Bradyrhizobium* isolates obtained from Córdoba, Argentina. Syst Appl Microbiol 34:446–452. doi:10.1016/j.syapm.2011.04.007. 21742454

[3] Chen J, Hu M, Ma H, Wang Y, Wang ET, Zhou Z, Gu J. 2016. Genetic diversity and distribution of bradyrhizobia nodulating peanut in acid-neutral soils in Guangdong Province. Syst Appl Microbiol 39:418–427. doi:10.1016/j.syapm.2016.06.002. 27499533

[4] Zazou AZ, Fonceka D, Fall S, Fabra A, Ibañez F, Pignoly S, Diouf A, Touré O, Faye MN, Hocher V, Diouf D, Svistoonoff S. 2018. Genetic diversity and symbiotic efficiency of rhizobial strains isolated from nodules of peanut (*Arachis hypogaea* L.) in Senegal. Agric Ecosyst Environ 265:384–391. doi:10.1016/j.agee.2018.06.001.

[5] Shao S, Chen M, Liu W, Hu X, Wang ET, Yu S, Li Y. 2020. Long-term monoculture reduces the symbiotic rhizobial biodiversity of peanut. Syst Appl Microbiol 43:126101. doi:10.1016/j.syapm.2020.126101. 32847777

[6] Jaiswal SK, Msimbira LA, Dakora FD. 2017. Phylogenetically diverse group of native bacterial symbionts isolated from root nodules of groundnut (*Arachis hypogaea* L.) in South Africa. Syst Appl Microbiol 40:215–226. doi:10.1016/j.syapm.2017.02.002. 28372899 PMC5460907

[7] Osei O, Abaidoo RC, Ahiabor BDK, Boddey RM, Rouws LFM. 2018. Bacteria related to *Bradyrhizobium yuanmingense* from Ghana are effective groundnut micro-symbionts. Appl Soil Ecol 127:41–50. doi:10.1016/j.apsoil.2018.03.003. 29887673 PMC5989812

[8] Vincent JM. 1970. A manual for the practical study of the root nodule bacteria. IBP handbook no. 15. Blackwell Scientific Publications, Oxford, United Kingdom.

[9] Edwards U, Rogall T, Blöcker H, Emde M, Böttger EC. 1989. Isolation and direct complete nucleotide determination of entire genes. Characterization of a gene coding for 16S ribosomal RNA. Nucleic Acids Res 17:7843–7853. doi:10.1093/nar/17.19.7843. 2798131 PMC334891

[10] Bolger AM, Lohse M, Usadel B. 2014. Trimmomatic: a flexible trimmer for Illumina sequence data. Bioinformatics 30:2114–2120. doi:10.1093/bioinformatics/btu170. 24695404 PMC4103590

[11] Nurk S, Bankevich A, Antipov D, Gurevich AA, Korobeynikov A, Lapidus A, Prjibelski AD, Pyshkin A, Sirotkin A, Sirotkin Y, Stepanauskas R, Clingenpeel SR, Woyke Y, McLean JS, Lasken R, Tesler G, Alekseyev MA, Pevzner PA. 2013. Assembling single-cell genomes and mini-metagenomes from chimeric MDA products. J Comput Biol 20:714–737. doi:10.1089/cmb.2013.0084. 24093227 PMC3791033

[12] Seemann T. 2014. Prokka: rapid prokaryotic genome annotation. Bioinformatics 30:2068–2069. doi:10.1093/bioinformatics/btu153. 24642063

[13] Danecek P, Bonfield JK, Liddle J, Marshall J, Ohan V, Pollard MO, Whitwham A, Keane T, McCarthy SA, Davies RM, Li H. 2021. Twelve years of SAMtools and BCFtools. Gigascience 10:giab008. doi:10.1093/gigascience/giab008. 33590861 PMC7931819

[14] Vasimuddin M, Misra S, Li H, Aluru S. 2019. Efficient architecture-aware acceleration of BWA-MEM for multicore systems, p 314–324. *In* IEEE International Parallel and Distributed Processing Symposium (IPDPS) (IEEE, 2019). IEEE, New York, NY. doi:10.1109/IPDPS.2019.00041.

[15] Chun J, Oren A, Ventosa A, Christensen H, Arahal DR, da Costa MS, Rooney AP, Yi H, Xu XW, De Meyer S, Trujillo ME. 2018. Proposed minimal standards for the use of genome data for the taxonomy of prokaryotes. Int J Syst Evol Microbiol 68:461–466. doi:10.1099/ijsem.0.002516. 29292687

[16] Mahato NK, Gupta V, Singh P, Kumari R, Verma H, Tripathi C, Rani P, Sharma A, Singhvi N, Sood U, Hira P, Kohli P, Nayyar N, Puri A, Bajaj A, Kumar R, Negi V, Talwar C, Khurana H, Nagar S, Sharma M, Mishra H, Singh AK, Dhingra G, Negi RK, Shakarad M, Singh Y, Lal R. 2017. Microbial taxonomy in the era of OMICS: application of DNA sequences, computational tools and techniques. Antonie Van Leeuwenhoek 110:1357–1371. doi:10.1007/s10482-017-0928-1. 28831610

